# The ameliorative effect of hemp seed hexane extracts on the *Propionibacterium acnes*-induced inflammation and lipogenesis in sebocytes

**DOI:** 10.1371/journal.pone.0202933

**Published:** 2018-08-27

**Authors:** Solee Jin, Mi-Young Lee

**Affiliations:** 1 Deparment of Medical Science, College of Medical Science, SoonChunHyang University, Asan, Chungnam, Korea; 2 Department of Medical Biotechnology, College of Medical Science, SoonChunHyang University, Asan, Chungnam, Korea; Zhejiang University College of Biosystems Engineering and Food Science, CHINA

## Abstract

In this study, we investigated the anti-microbial, anti-inflammatory, and anti-lipogenic effects of hemp (*Cannabis sativa L*.) seed hexane extracts, focusing on the *Propionibacterium acnes*-triggered inflammation and lipogenesis. Hemp seed hexane extracts (HSHE) showed anti-microbial activity against *P*. *acnes*. The expression of iNOS, COX-2, and the subsequent production of nitric oxide and prostaglandin increased after infection of *P*. *acnes* in HaCaT cells, however, upon treating with HSHE, their expressions were reduced. *P*. *acnes*-induced expressions of IL-1β and IL-8 were also reduced. HSHE exerted anti-inflammatory effects by regulating NF-κB and MAPKs signaling and blunting the translocation of p-NF-κB to the nucleus in *P*. *acnes*-stimulated HaCaT cells. Moreover, *P*. *acnes*-induced phosphorylation of ERK and JNK, and their downstream targets c-Fos and c-Jun, was also inhibited by HSHE. In addition, the transactivation of AP-1 induced by *P*. *acnes* infection was also downregulated by HSHE. Notably, HSHE regulated inflammation and lipid biosynthesis via regulating AMPK and AKT/FoxO1 signaling in IGF-1-induced inflammation and lipogenesis of sebocytes. In addition, HSHE inhibited 5-lipoxygenase level and *P*. *acnes*-induced MMP-9 activity, and promoted collagen biosynthesis *in vitro*. Thus, HSHE could be utilized to treat acne vulgaris, through its anti-microbial, anti-inflammatory, anti-lipogenic, and collagen-promoting properties.

## Introduction

Hemp (*Cannabis sativa L*.), also known as cannabis, has been an important plant used in dietary supplements, clothing, cosmetics, and medicines [1,2]. Hemp has also been used as a medicament for the treatment of medical conditions such as rheumatic pain, intestinal constipation, disorders of the female reproductive system, and malaria [3]. However, due to its hallucinogenic effects, the use of cannabis has been legally restricted in almost all countries of the world, except for some countries, such as the US that partially allow it for medicinal use. In US, with a doctor's recommendation, the medical use of cannabis is legal in 29 states, the District of Columbia, and the territories of Guam and Puerto Rico. Seventeen other states allow the medical use of cannabis with low THC/high CBD. Tetrahydrocannabinol (THC) is the major psychoactive component of cannabis, even small amounts of which could be highly hallucinogenic, while cannabidiol (CBD) is the non-psychoactive component of cannabis. THC binds cannabinoid CB1 and CB2 receptors with high affinity and activates them, while CBD has a very low affinity for both CB1 and CB2 receptors, and acts as an indirect antagonist of these receptors [1,4]. THC is effective for relieving pain, inhibiting inflammation, and relaxing muscle tension. Thus, it can be used for patients with multiple sclerosis, neurodegenerative disorders, and severe pain. However, overdose of THC may cause serious mental side effects of hallucinations [5,6]. On the contrary, hemp seed does not have any psychotropic chemical components. Moreover, hemp is the only seed plant without any saturated fatty acids, containing mostly polyunsaturated fatty acids including linoleic acid and gamma-linolenic acid [7].

Acne vulgaris is observed in 85% of adolescents and refers to a disease that causes inflammation of sebaceous glands attached to hairfollicles. *Propionibacterium acnes* (*P*. *acnes*), present on normal skin, activates an immune response and changes the lipid composition of the sebaceous glands [8,9]. *P*. *acnes* stimulates the inflammatory cytokines such as interleukin (IL)-1β, IL-8, and leukotrienes by activating Toll-like receptors (TLRs) and induces the expression of enzymes such as inducible nitric oxide synthase (iNOS) and cyclooxygenase-2 (COX-2), causing chronic inflammation [10–12]. It also activates the nuclear factor kappa B (NF-κB) and mitogen-activated protein kinases (MAPKs) signals, and upregulates related genes to carry out innate immunity.

Keratinocytes and dermal fibroblasts secrete matrix metalloproteinases (MMPs) which mediate the digestion of many components of extracellular matrix (ECM) in response to external stimuli such as bacterial infection, oxidative stress, and UV radiation. MMPs are classified into five main subgroups, such as collagenases, gelatinases, stromelysins, matrilysins, and 5 membrane-type MMPs, based on their structure and substrate specificity. MMP-1 is a collagenase that digests fibrillar collagen via recognition of a hemopexin-like domain, while MMP-2 and MMP-9 are gelatinases that degrade a number of ECM components, such as type I and IV collagen [13,14].

Human sebocytes are specialized sebum-producing epithelial cells that produce and release lipid droplets. Sebum is an integral component of the epidermal barrier and its formation is involved in the skin immune system. Excess sebum production causes acne by inducing an inflammatory reaction under the proliferating skin microflora [15]. Peroxisome proliferator-activated receptor gamma (PPARγ) present in sebaceous gland cells regulates the lipid production and lipid metabolism via modulation of the AMP-activated protein kinase (AMPK) signal. AMPK is a serine/threonine kinase that plays a regulatory role in glucose and lipid metabolism [16]. Activation of AMPK by phosphorylation down-regulates the expression of fatty acid synthase (FAS) and sterol regulatory element-binding protein 1 (SREBP-1), by inhibiting phosphorylation of mTOR [17,18]. Upon activation by insulin-like growth factor-1 (IGF-1), protein kinase B (AKT) phosphorylates fork head box protein O1 (FoxO1) to enhance lipogenesis. FoxO1 is reported to directly bind and modulate PPAR-γ function [19]. Additionally, 5-lipoxygenase induces the release of leukotriene (LT)-B_4_, a pro-inflammatory lipid, which promotes lipid synthesis in acne lesions. Thus, inhibition of 5-lipoxygenase may be an ideal target for downregulation of inflammation and lipid accumulation in sebocytes [20,21].

Hemp seeds have been primarily used in nutraceutical and pharmaceutical industries, utilizing their health-promoting properties and ideal nutrient content. However, information on its anti-acne activity and underlying mechanism is limited. In this study, the ameliorative effects of hemp seed hexane extracts on *P*. *acnes*-induced inflammation in HaCaT cells and IGF-1-stimulated lipogenesis in sebocytes were examined at the molecular and cellular level.

## Materials and methods

### Preparation of hemp seed hexane extracts

Hemp seeds imported from Canada were purchased from an online Korean store called Nooriwon. The dried seeds were ground and soaked with three volumes of hexane for 24 h while stirring. The extract was serially filtered using a No. 2 filter and a nylon filter (0.45 μm) (both from Whatman International Ltd., UK). The extraction procedure was repeated twice with the residue. The solvent was evaporated using a rotary vacuum evaporator (Eyela, Japan) at 35°C. Residual solvent was re-evaporated twice at 70°C for 10 min. To increase the solubility of HSHE in the cell culture media, same volume of dimethyl sulfoxide (DMSO) was added to the hexane-extracted hemp seed oil and mixed by vortexing at room temperature. All experiments were performed under conditions that did not show toxicity by hemp seed hexane extract and DMSO [22].

### Microbial cultivation and anti-acne activity determination

*Propionibacterium acnes* (*P*. *acnes*, KCTC) was obtained from the Korean Culture Center of Microorganisms, Seoul, Korea. *P*. *acnes* was grown anaerobically in solid and liquid Reinforced Clostridial Medium (RCM) at 37°C for 72 h. *P*. *acnes* were harvested via centrifugation of the cultures at 4,500 rpm for 15 min at 4°C. The resulting bacterial pellets were pooled, washed in cold 1× PBS, and centrifuged again. Finally, the pooled bacterial pellets were resuspended in serum free medium (bacterial concentration was 1.2X10^9^ CFU/mL). The suspension was heated at 80°C and the heat-killed bacteria were used for the stimulation experiments reported previously by us [9].

To determine anti-acne activity of the HSHE, the culture of *P*.*acnes* was standardized using microbiological No. 0.5 McFarland Standard’s solution according to the recommendations of the Clinical Laboratory Standards Institute (CLSI) [23]. The anti-microbial effect of HSHE was measured on agar medium. 200 μL cell suspension (10^5^ cells/mL) was mixed with 100 μL of HSHE diluted in RCM medium at 37°C for 30 min. The control was mixed with 100 μL medium and reacted under the same conditions. Then, 100 μL of each mixture was spread on the surface of RCM medium on a petri dish. Both control and experimental groups were incubated at 37°C for 72 h.

### Cell culture

The human keratinocyte (HaCaT) and human fibroblast cell lines (Hs68) were obtained from the American Type Culture Collection (ATCC). The cells were incubated in complete Dulbecco’s modified Eagle’s medium (DMEM; Hyclone, Logan, UT, USA) containing 100 U/mL penicillin, streptomycin (100 μg/mL), and 10% fetal bovine serum (FBS) at 37°C [24,25]. Primary human sebocytes (Celprogen Inc., USA) were maintained in Human Sebocyte Complete Growth Media purchased from the same vendor. For HSHE treatment, cells were seeded and incubated overnight prior to the treatments. For the stimulation experiment, HaCaT and Hs68 cells were incubated with heat-killed *P*. *acnes* adjusted at the appropriate concentration in serum free media for 24 h at 37°C in 5% CO_2_. After stimulation, the HaCaT cells were treated with or without hemp seed hexane extracts for 24 h at 37°C in 5% CO_2_. Sebocytes were pre-treated with or without hemp seed hexane extracts for 2 h. Then, 120 ng/mL IGF-1 was added to each plate and the cells were incubated for 22 h [9].

### NO production measurement

The nitrite concentration in conditioned medium was measured as an indicator of NO production according to the Griess reaction. Each supernatant (100 μL) was mixed with the same volume of Griess reagent (1% sulfanilamide in 5% phosphoric acid and 0.1% naphthyl ethylenediamine dihydrochloride in distilled water). The absorbance of the mixture was determined with an ELISA reader (Sunrise, Tecan, Switzerland) at 570 nm [9, 26].

### Enzyme-Linked Immunosorbent Assay (ELISA)

The effects of HSHE on IL-8 and prostaglandin E_2_ (PGE_2_) productions in heat-killed *P*. *acnes*-infected HaCaT cells and on the 5-lipoxygenase production in sebocytes, stimulated with IGF-1, were measured with ELISA [27]. The IL-8 (BD Science, USA), PGE_2_ (LSBio, USA) and 5-lipoxygenase (MyBioSource, USA) concentrations were calculated according to the standard curve using standard in the ELISA kit.

### Western blot analysis

Proteins were separated by 10% SDS-PAGE, and transferred onto polyvinylidene fluoride membrane (Bio-Rad Laboratories, CA) [28]. After blocking for 2 h at room temperature, the membranes were incubated overnight at 4°C with primary antibodies against iNOS, IL-1β, COX-2, IKKα, IKKβ, p-IKKα/β, IκBα, p-IκBα, NF-κB p65, p-NF-κB p65, p38, p-p38, ERK, p-ERK, JNK, p-JNK, AKT, p-AKT, mTOR, p-mTOR, PPARγ, FAS, AMPKα, p-AMPKα, FoxO1, and p-FoxO1 (Cell Signaling Technology, USA), SREBP1 (Novus Biologicals, Canada) and β-actin (Santa Cruz Biotechnology, USA) which were diluted using manufacturers’ recommendations [25,29]. The membranes were then washed in 1× TBST and incubated with the appropriate secondary antibody HRP-conjugated (1:5000) at room temperature for 1 h. Protein bands were visualized using the Sensi-Q 2000 (Lugen, South Korea). The intensity of the bands was analyzed using ImageJ and normalized against β-actin [30].

### Transient transfection and luciferase assay

The effects of HSHE on AP-1 activity were assayed in a Luciferase Reporter Assay System (Promega, Madison, WI, US) [8]. Upon reaching 60–70% confluency, the HaCaT cells were washed in PBS and the cells were then transfected with AP-1-Luc reporter vector (Affymetrix, Santa Clara, CA, US) using Fugene 6 (Promega, Madison, WI,US), according to the manufacturer’s protocol. After the 24 h transfection, cells were infected with *P*. *acnes*, medium was changed, and treated with various concentrations of hemp seed hexane extracts. The processed cells were cultured for an additional 24 h. Cells were washed with PBS, lysed with lysis reagent, and treated with the luciferase assay substrate. Luciferase activity was determined using the luminometer (Infinite F200 PRO, Tecan, Männedorf, Switzerland).

### Confocal microscope analysis and gelatin zymography

Confocal microscopic analysis for NF-κB translocation and gelatin zymographic analysis of MMP activity on Hs68 cells was performed as described in our previous report [9].

### Collagen synthesis-promoting assay

Collagen contents in the ECM of Hs68 cells were determined by Sircol collagen assay (Biocolor, UK) according to the manufacturer's protocols. Collagen dye complexes formed in cell supernatants were added to the Sircol staining reagent and precipitated in soluble non-binding dyes. After centrifugation, the pellet was washed with ice-cold acid-salt wash reagent and reacted with alkali reagent. Samples were dispensed into a 96-well plate and the absorbance was read at 570 nm. The amount of collagen was calculated based on a standard curve obtained with the standard bovine type I collagen supplied with the kit [31].

### Data analysis

Data was analyzed using the IBM Statistical Package for Social Sciences (SPSS, version 20). All the data were presented as mean ± standard deviation (SD) of triplicate experiments. One-way analysis of variance (ANOVA) with a Duncan multiple-comparison test was utilized to determine the statistical differences among groups. *P*-values <0.05 were considered statistically significant [32].

## Results

### Anti-microbial effect of hemp seed hexane extracts on *P*. *acnes*

**Fig 1:**
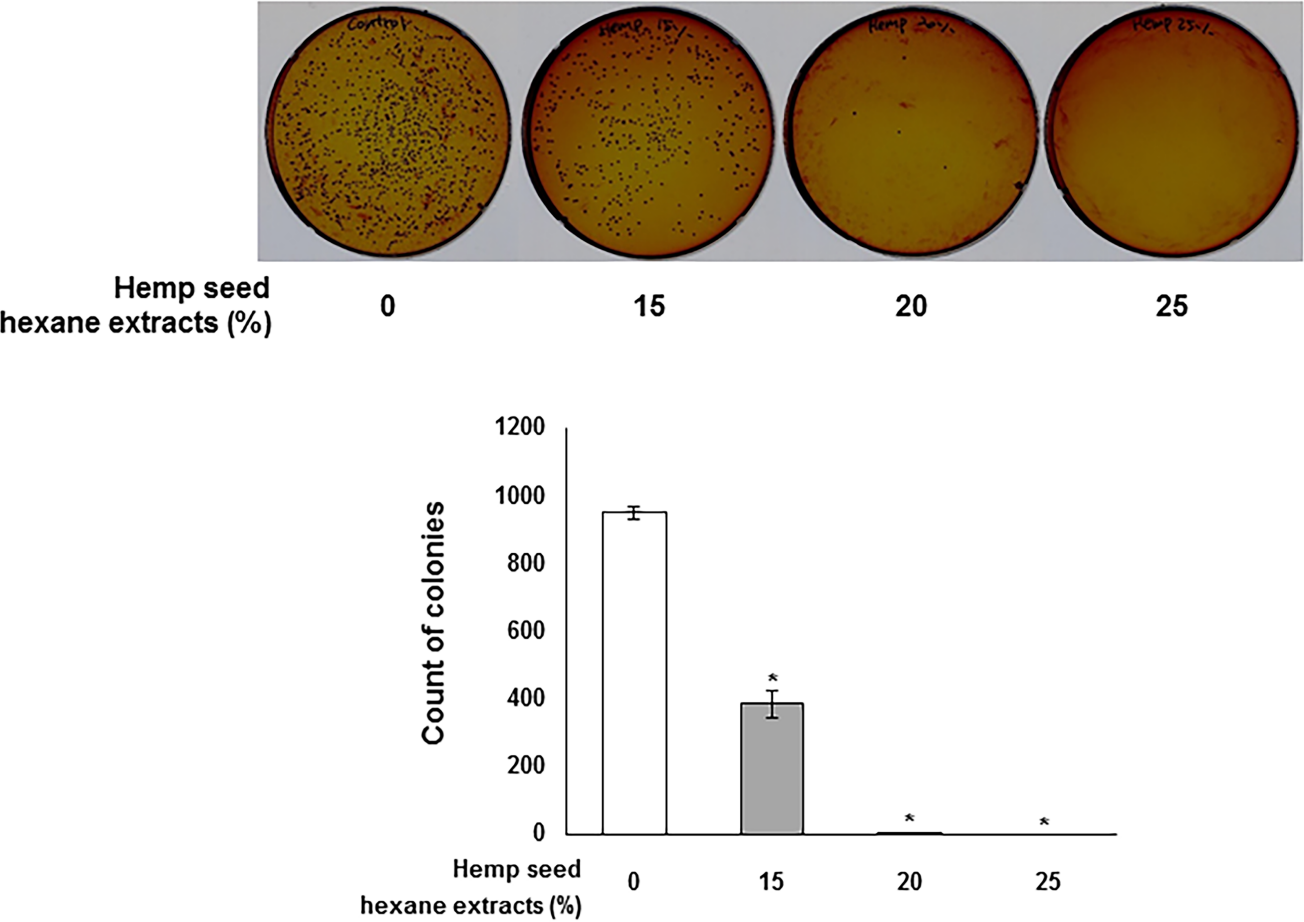
Effect of hemp seed hexane extracts against the growth of *P*. *acnes*. All data are presented as mean±SD of three independent experiments. *p<0.05 indicates significant differences compared to control group.

At first, we investigated the anti-microbial effect of HSHE against *P*. *acnes* (Fig 1). *P*. *acnes* was treated with 0, 15, 20 and 25% HSHE and the number of colonies was counted to examine anti-microbial activity against *P*. *acnes* of HSHE. 15 and 20% HSHE showed approximately 59% and 99% of anti-microbial activity compared to control. At 20% HSHE, complete inactivation of *P*.*acnes* was observed. Erythromycin (3 ppm), a conventional anti-microbial agent for acne, showed approximately 67% anti-microbial activity (Data not shown). These results suggest that HSHE is able to inactivate the growth of *P*. *acnes*.

### Inhibitory effect of HSHE on *P*. *acnes*-induced inflammation in HaCaT cells

**Fig 2:**
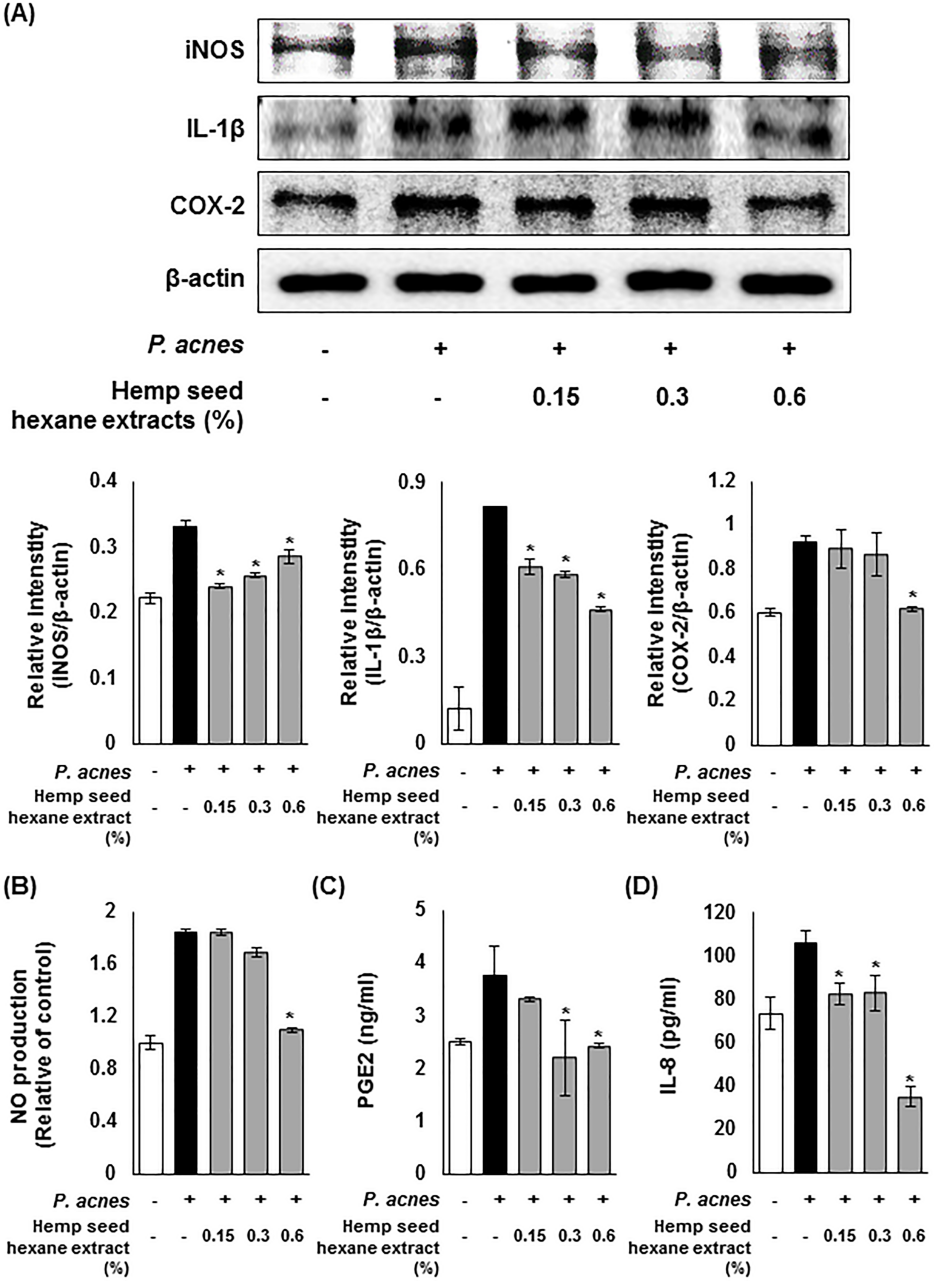
Inhibitory effects of hemp seed hexane extracts on *P*. *acnes-*induced inflammation in HaCaT cells. (A) Effects of hemp seed hexane extracts (HSHE) on iNOS, COX-2 and IL-1β expression in *P*. *acnes*-stimulated HaCaT cells. The expressions of iNOS, COX-2, and IL-1β were analyzed with ImageJ and normalized against β-actin. (B) Effects of HSHE on NO production in *P*. *acnes*-stimulated HaCaT cells. ELISA results demonstrate that HSHE reduced PGE_2_ (C) and IL-8 (D) in *P*. *acnes*-infected HaCaT cells. ELISA results demonstrate that HSHE reduced in *P*. *acnes*-infected HaCaT cells. All data are expressed as mean ± SD. *P<0.05 compared with *P*. *acnes* treated cells only.

To investigate the effect of HSHE on the expression of inflammatory enzymes iNOS and COX-2, and pro-inflammatory cytokine IL-1β, western blotting was performed with *P*.*acnes*-infected HaCaT cells. As shown in Fig 2A, the expression of iNOS and COX-2 was enhanced upon the stimulation of *P*. *acnes*, however, HSHE down-regulated the induced expression of iNOS and COX-2. Moreover, the expression of *P*.*acnes*-induced IL-1β was significantly inhibited at 0.6% HSHE. In addition, HSHE significantly suppressed the production of NO caused by iNOS in *P*. *acnes*-infected HaCaT cells in a dose dependent manner (Fig 2B). At 0.6% HSHE, about 40% of NO production was inhibited, compared to the *P*. *acnes*-induced inflammation group. Prostaglandin E_2_ (PGE_2_), which was generated by COX-2, was also notably inhibited by HSHE (Fig 2C). The ELISA results showed that there was a reduction in pro-inflammatory cytokine IL-8 secretion caused by HSHE (Fig 2D). These results indicate that HSHE exert anti-inflammatory activity via regulating inflammation-related enzymes, their products, and pro-inflammatory cytokine secretion.

**Fig 3:**
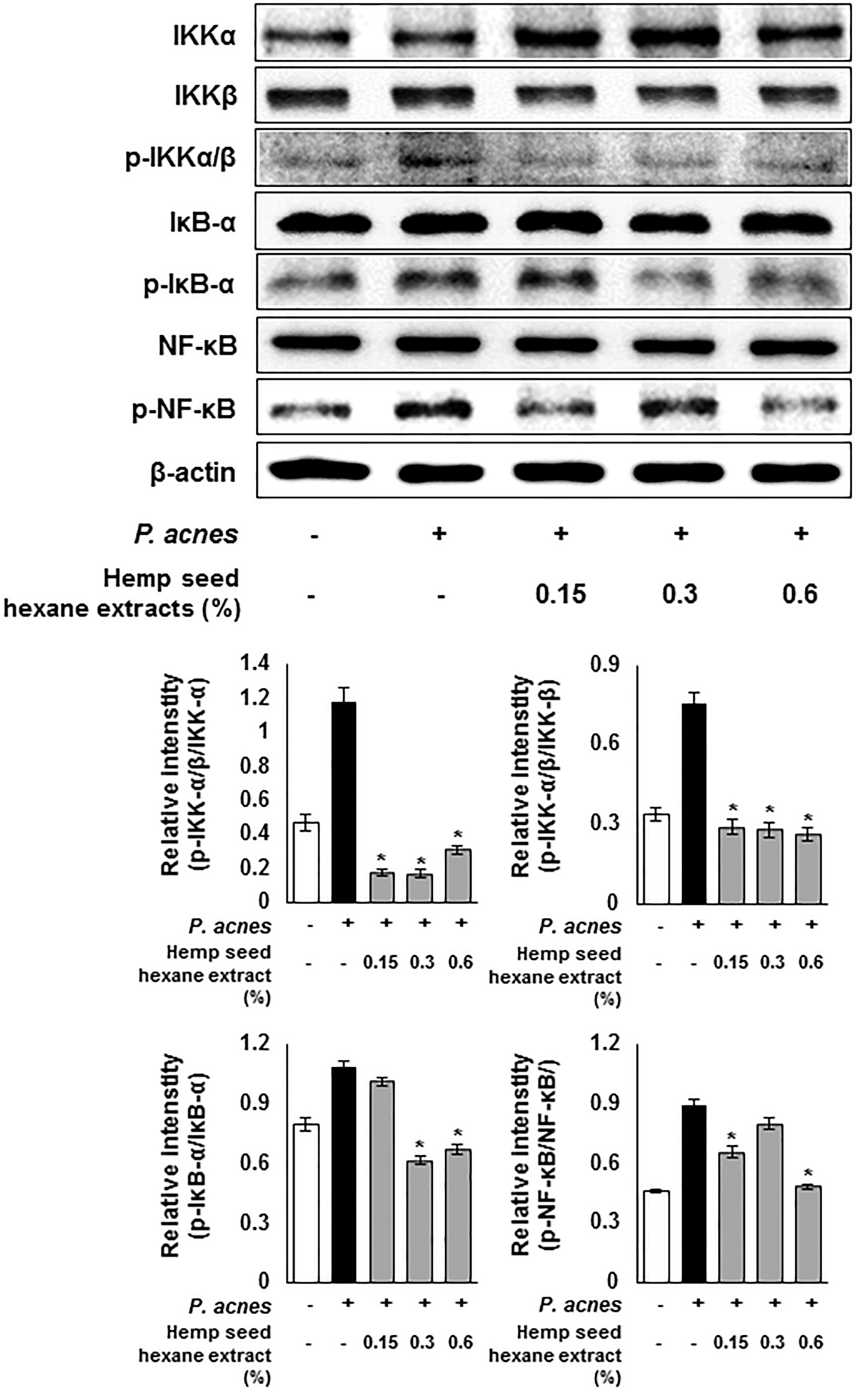
Hemp seed hexane extracts effectively inhibit the NF-κB signaling pathway in *P*. *acnes*-treated HaCaT cells. The expression of p-IKKα/β, p-IκB-α and p-NF-κB were analyzed with ImageJ and normalized against β-actin. Results are expressed as mean ± SD. *P<0.05 compared with *P*. *acnes* treated cells only.

**Fig 4:**
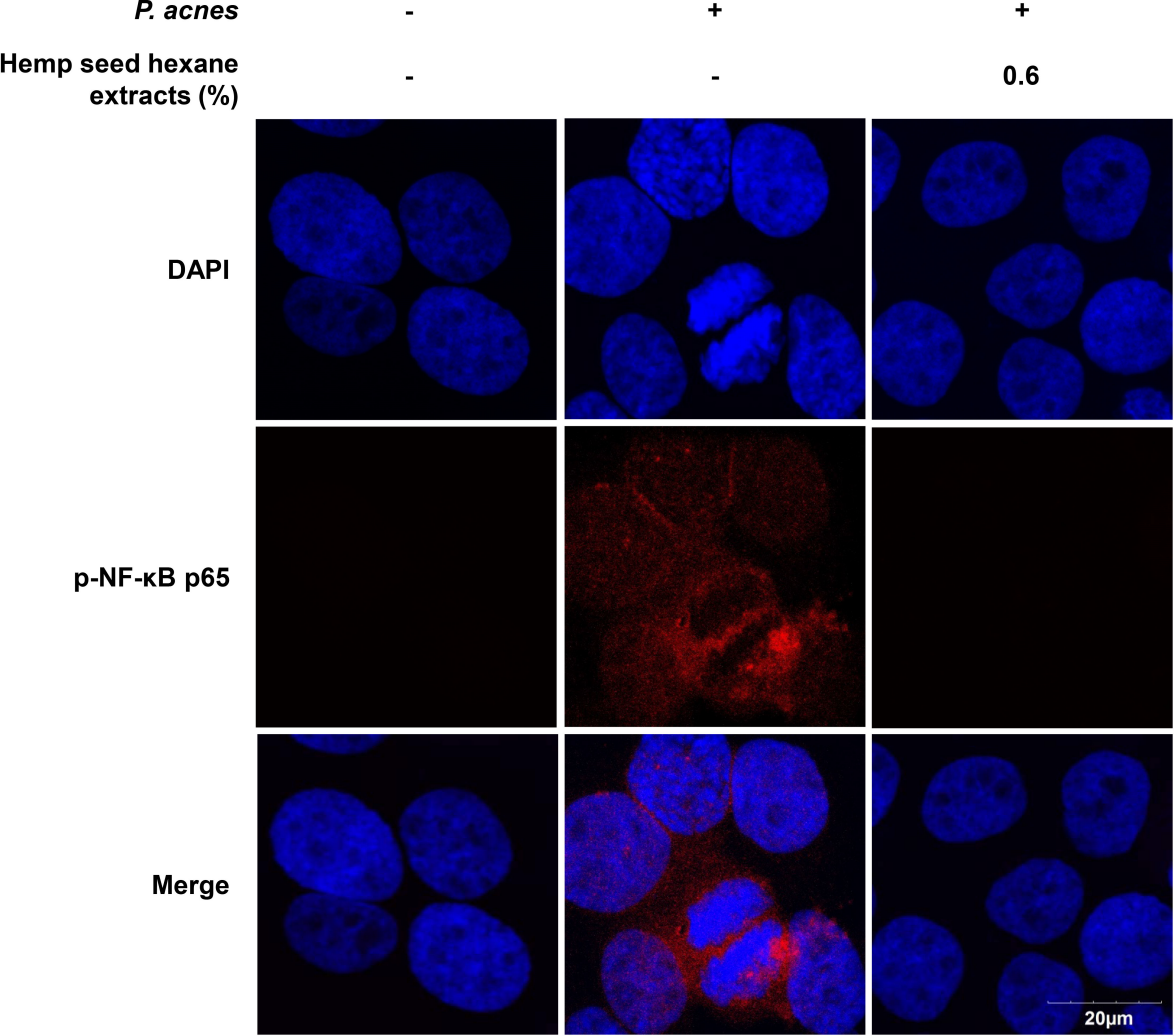
Effect of hemp seed hexane extracts on p-NFκB p65 nuclear translocation in *P*. *acnes*-induced HaCaT cells. Immunofluorescence staining for p-NF-κB (red) in *P*. *acnes*-exposed HaCaT cells without and with 0.6% HSHE. Nuclei are stained with DAPI (blue). HSHE reduced the nuclear translocation and accumulation of p-NF-κB, which was induced by *P*. *acnes*. Scale bar = 20 μm.

Next, the anti-inflammatory effect of HSHE was investigated based on the NF-κB signaling pathway (Fig 3). The expression levels of *P*.*acnes*-induced phosphorylated p-IKKα/β, p-IκB-α and p-NF-κB were investigated in *P*. *acnes*-stimulated HaCaT cells. The expression of *P*.*acnes*-induced phosphorylated IKKα/β, IκBα and NF-κB was significantly down-regulated by the action of HSHE. These results suggest that anti-inflammatory activity of HSHE was involved in suppression of NF-κB signaling pathway in *P*. *acnes*-stimulated HaCaT cells. In addition, suppression of nuclear translocation of p-NF-κB in *P*. *acnes*-infected HaCaT cells by HSHE was observed via confocal microscopy (Fig 4). The nuclear translocation and accumulation of p-NF-κB in *P*. *acnes*-infected HaCaT cells were dramatically reduced upon treatment with HSHE. These results suggest that HSHE exerts anti-inflammatory effects by regulating the NF-κB signaling pathway and inhibiting p-NF-κB nuclear translocation.

**Fig 5:**
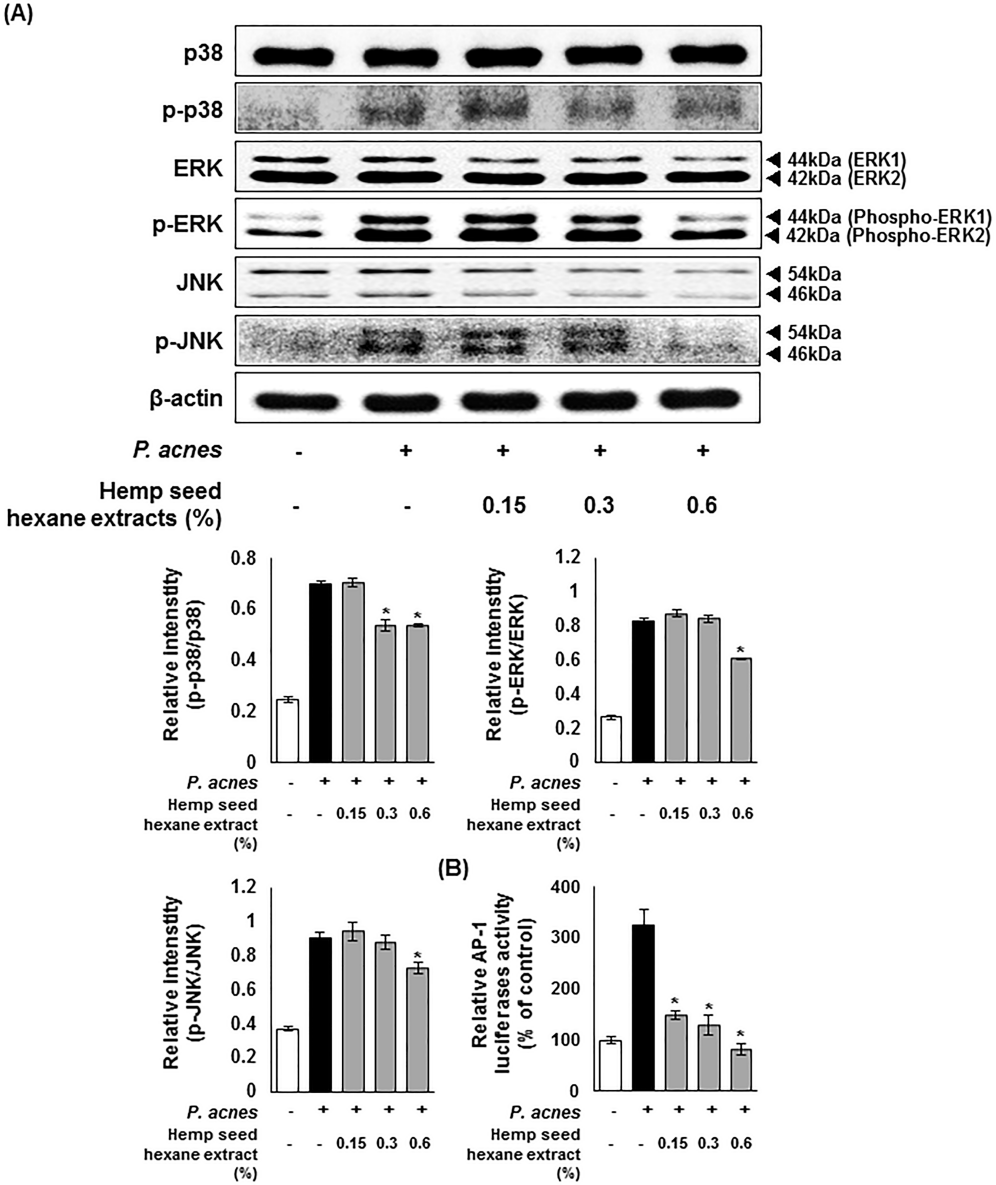
Hemp seed hexane extracts effectively inhibit the MAPK signaling pathway in *P*. *acnes*-treated HaCaT cells. (A) The expression of p-p38, p-ERK, and p-JNK were analyzed with ImageJ and normalized against β-actin. (B) Transcriptional activation of AP-1 was analyzed by luciferase reporter assay. AP-1 signaling was down-regulated by HSHE in HaCaT cells. Results are expressed as mean ± SD. *P<0.05 compared with *P*. *acnes* treated cells only.

Fig 5A shows the expression pattern of MAPK-related proteins in the presence of HSHE. As shown in the western blot analysis, the expression of phosphorylated p38, extracellular signal-regulated kinase (ERK), and c-Jun N-terminal kinase (JNK) elicited by *P*.*acnes* infection was significantly decreased upon HSHE treatment. The data indicate that HSHE inhibits phosphorylation of p38, ERK, and JNK, which are the major molecules of the MAPK signaling cascade, causing an anti-inflammatory response. *P*. *acnes* infection induces the formation of ROS in keratinocytes [14]. ROS generation in turn promotes the production of mitogen-activated protein kinase (MAPK) cascades including ERK and JNK, which subsequently regulate the activator protein 1 (AP-1) [33,34]. Increased AP-1 activity leads to MMPs activation and type I procollagen reduction [8]. The transcriptional activity of AP-1 relies on c-Jun phosphorylation and c-Fos expression [34–36]. Fig 5B shows the luciferase assay data; *P*. *acnes*-induced AP-1 promoter activity in HaCaT cells was decreased with HSHE, in a dose dependent manner. Approximately 54% of the AP-1 promoter activity was inhibited by 0.15% HSHE, and in particular 0.6% HSHE treatment showed almost similar expression levels as the control.

### Stimulatory effect of HSHE on *in vitro* collagen synthesis

**Fig 6:**
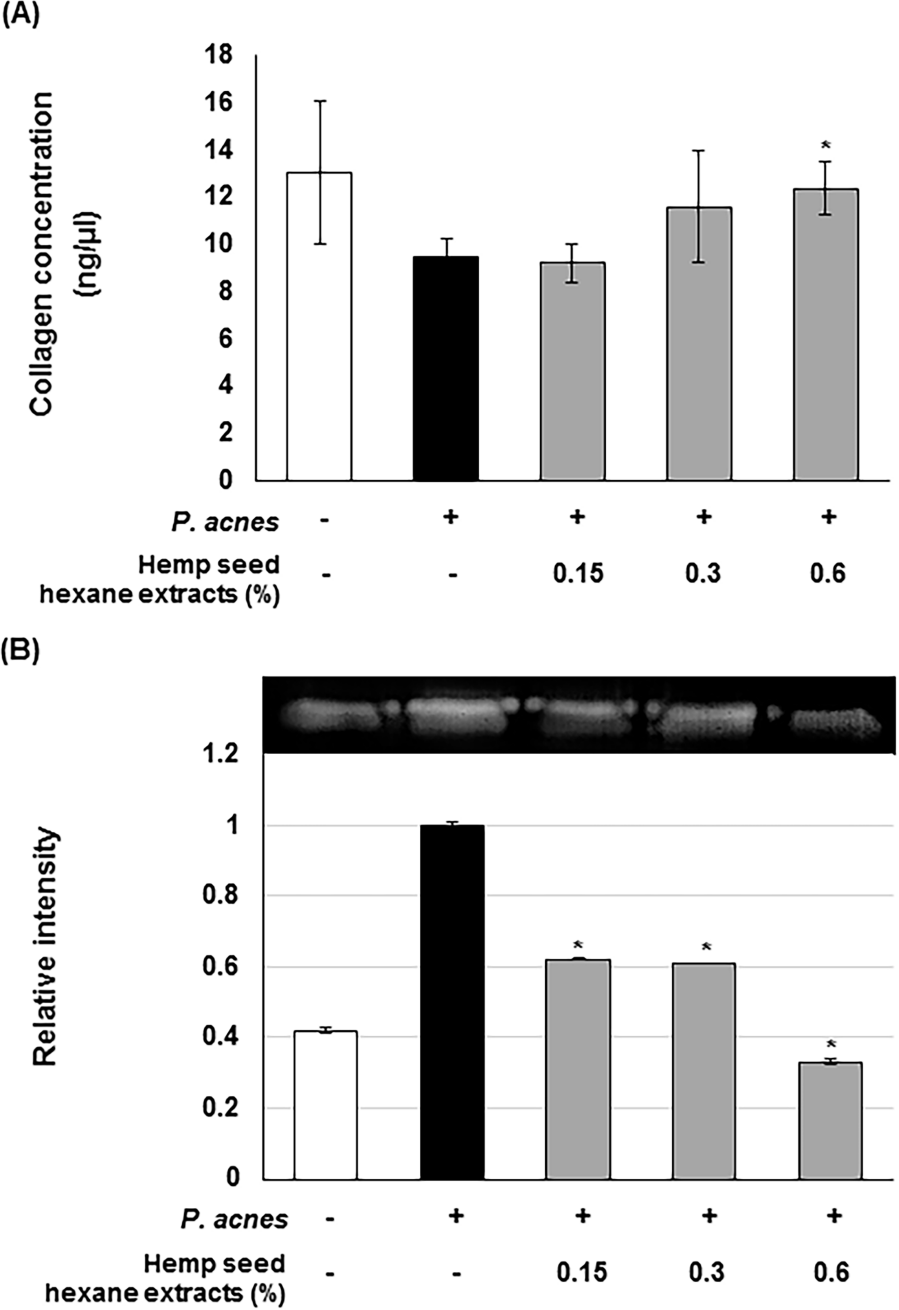
Stimulatory effect of hemp seed hexane extracts on *in vitro* collagen synthesis. (A) Effect of HSHE on collagen production in *P*. *acnes*-induced Hs68 cells. (B) Effect of HSHE on *P*. *acnes*-induced MMP-9 activity (gelatinase) on zymographic gel. The expression of MMP-9 was analyzed with ImageJ and normalized against *P*. *acnes* treated cells only. All data are expressed as mean ± SD. *P<0.05 compared with *P*. *acnes* treated cells only.

Collagen biosynthesis plays a critical role in wound healing and skin regeneration. To investigate the effects of HSHE on collagen production in *P*.*acnes*-infected dermal fibroblast, the collagen levels were assessed in the supernatant of *P*.*acnes*-infected Hs68 cells. As shown in Fig 6A, HSHE increases collagen production, compared to the group infected with *P*.*acnes* alone, which shows its potential to improve wound healing and skin regeneration processes. Moreover, HSHE inhibited the MMP-9 activity in *P*.*acnes*-infected Hs68 cells, as shown in gelatin zymography (Fig 6B). About 40% of MMP-9 activity was inhibited compared to the *P*. *acne*-treated Hs68 cells at 0.15% HSHE. These results suggest that HSHE is able to reduce ECM damage caused by *P*. *acnes* infection.

### Inhibitory effects of HSHE on lipogenic signals in sebocytes

**Fig 7:**
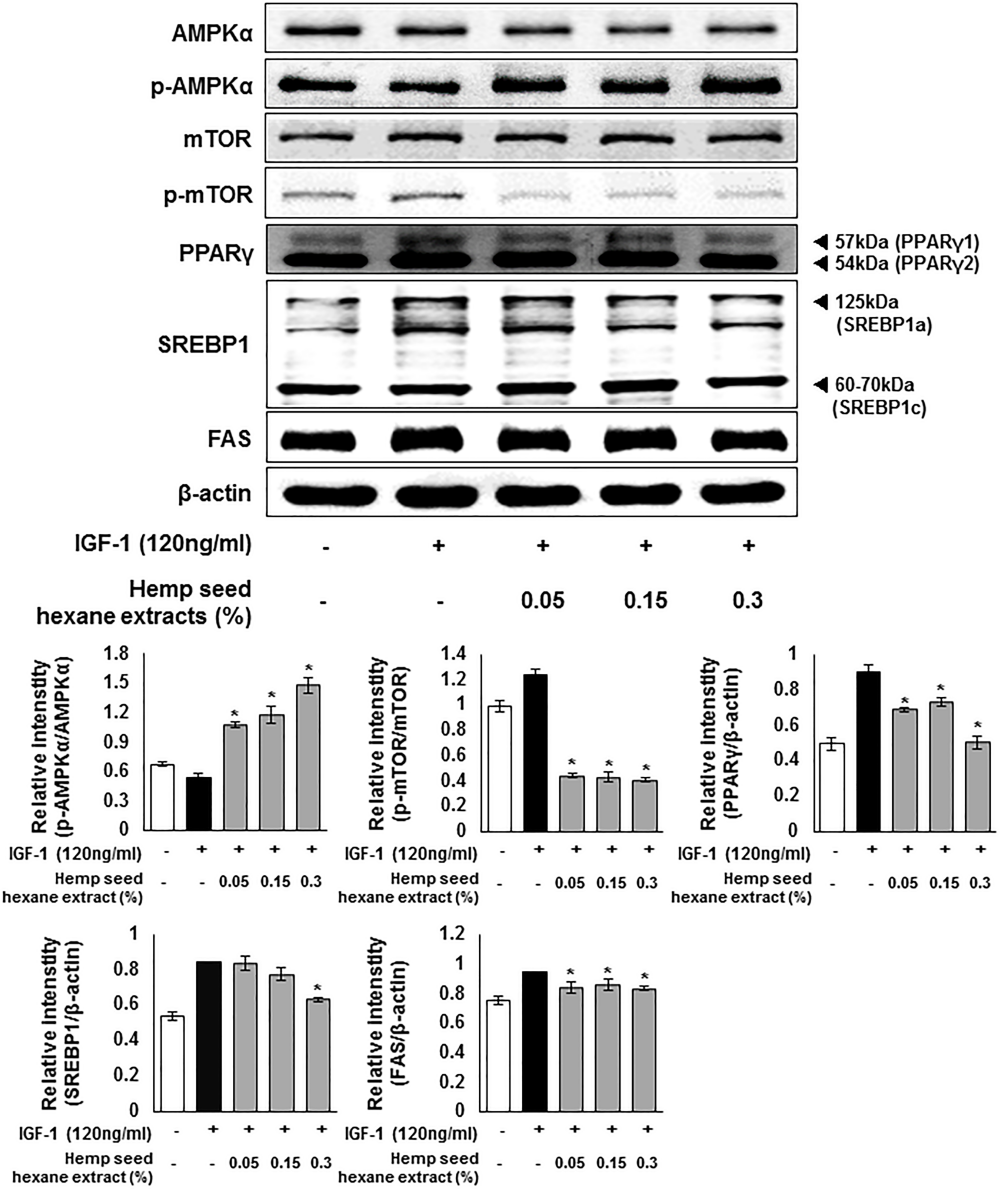
Regulation of intracellular lipogenesis through AMPK signals in IGF-1-induced sebocytes. The expression of p-AMPKα, p-mTOR, SREBP1, and FAS was analyzed with ImageJ and normalized against β-actin. Results are expressed as mean ± SD. *P<0.05 compared with IGF-1 treated cells only.

To investigate the anti-lipogenic effects of HSHE exerted via regulating lipogenic signaling pathway, IGF-1 was treated in order to trigger the lipid production in cultured human sebocytes. As indicated in Fig 7, upon stimulation by IGF-1, the expression of p-AMPKα was slightly reduced, whereas the expression levels of p-mTOR, PPARγ, SREBP1, and FAS increased. However, addition of HSHE enhanced the expression of p-AMPKα, but the extracts reduced the expression of p-mTOR, PPARγ, SREBP1, and FAS. These results indicate that HSHE has a suppressive effect on intracellular lipid synthesis in sebocytes.

**Fig 8:**
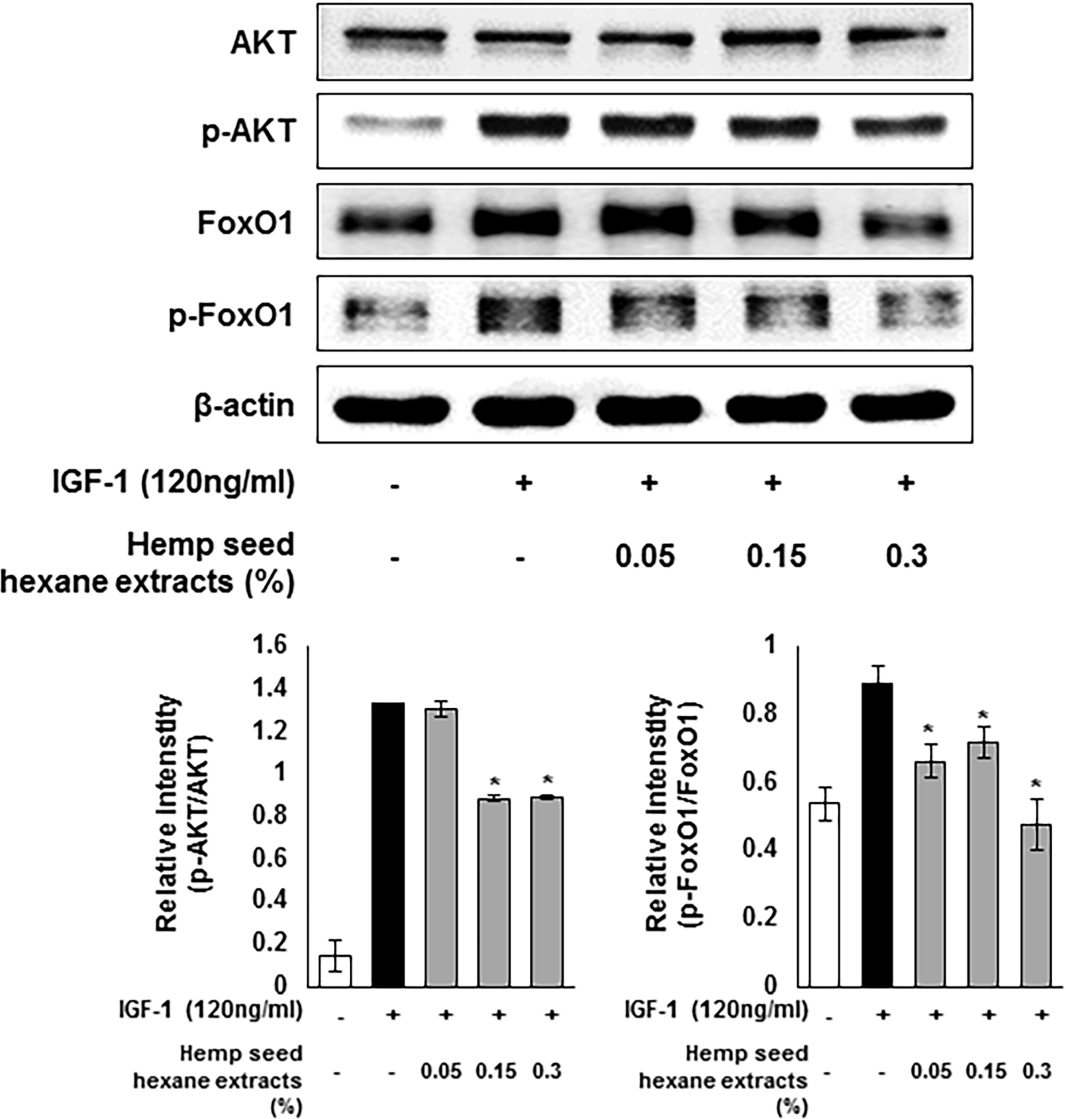
Regulation of intracellular lipogenesis through the AKT/FoxO1 signals in IGF-1-induced sebocytes. The expression levels of p-AKT and p-FoxO1 were analyzed with ImageJ and normalized against β-actin. Results are expressed as mean ± SD. *P<0.05 compared with IGF-1 treated cells only.

Moreover, HSHE regulated the intracellular lipogenesis through the AKT/FoxO1 signaling as shown in Fig 8. The Akt pathway, which is downstream of the insulin signaling, plays a crucial role in adipocyte differentiation. Phosphorylation of FoxO1 is mediated by p-AKT, thereby modulating PPARγ activity to induce lipogenesis [16]. The expression of phosphorylated AKT and FoxO1 was increased in IGF-1-treated sebocytes, however, HSHE inhibited the phosphorylation of AKT and FoxO1; suggesting down-regulation of intracellular lipid production.

**Fig 9:**
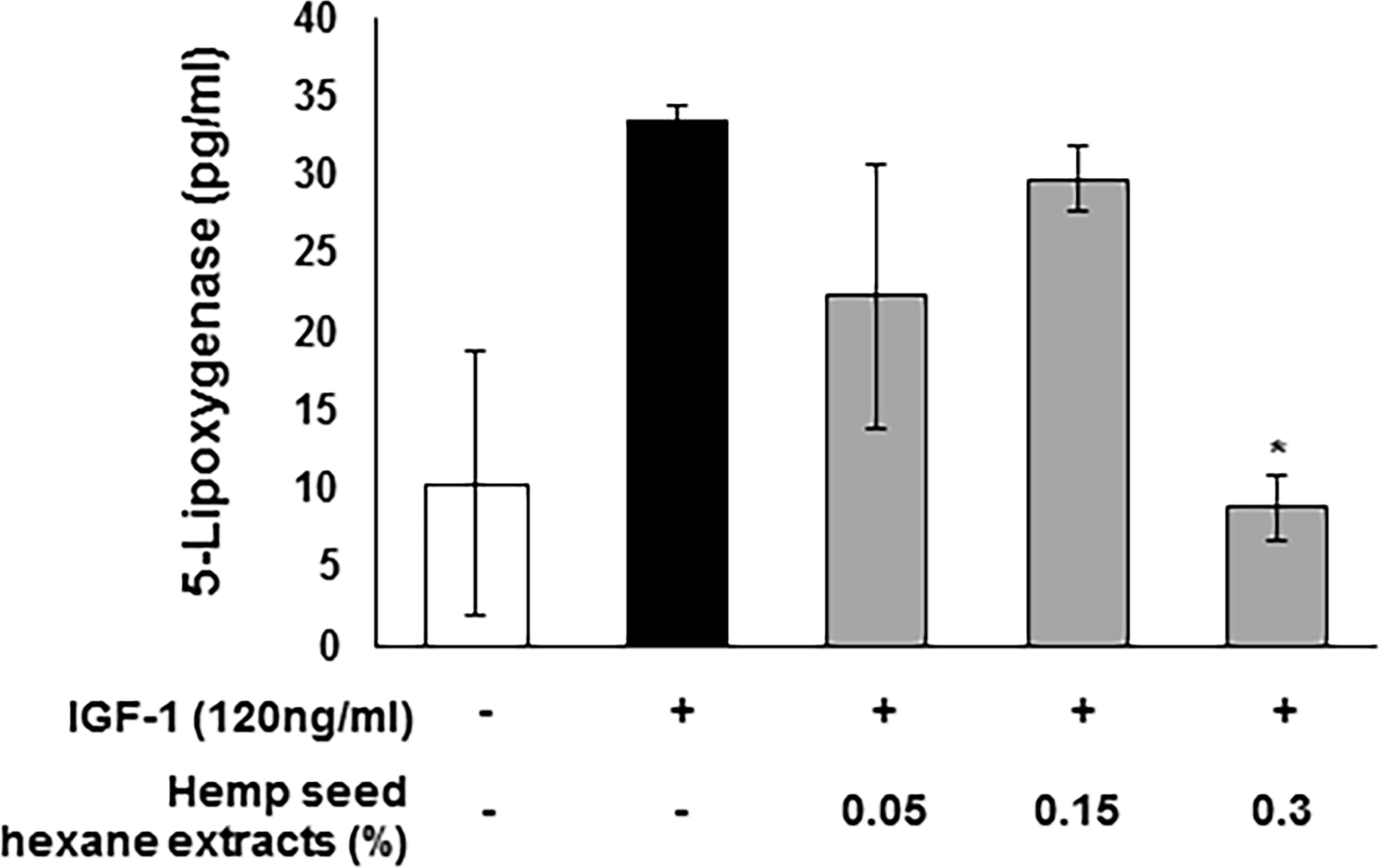
Inhibitory effect of hemp seed hexane extracts on the IGF-1-stimulated 5-lipoxygenase level in sebocytes. All data are expressed as mean ± SD. *P<0.05 compared with IGF-1 treated cells only.

5-lipoxygenase promotes the inflammatory response as well as lipid synthesis in the sebaceous glands through leukotriene (LT)-B_4_ production catalyst [20]. Approximately 73% 5-lipoxygenase was inhibited at 0.3% HSHE treatment compared to the group treated with IGF-1 alone (Fig 9). Therefore, HSHE might down-regulate inflammation and lipid synthesis in sebaceous glands by reducing the 5-lipoxygenase levels.

## Discussion

Acne vulgaris is a chronic inflammatory disease with multifactorial pathogenesis, though the mechanisms underlying the pathogenesis and subsequent development are not yet completely elucidated. Inflammation and lipogenesis are the major factors involved in acne onset. The factors that regulate inflammation and sebum production are being extensively studied in order to identify novel therapeutic strategies for acne treatment.

*P*. *acnes* directly causes inflammation of keratinocytes [37]. *P*. *acnes*-infected keratinocytes activate TLRs and induce the secretion of inflammatory cytokines and chemokines such as TNF-α, IL-1β, and IL-8 through the expression of inflammatory enzymes. The activity of TLRs also activates the NF-κB and MAPK signaling processes [38], and the activated NF-κB then enters the nucleus, inducing gene transcription involved in the inflammatory responses. When excess sebum is produced in the sebaceous glands, symbiotic skin microbes including *P*. *acnes* easily grow and activate inflammation in acne lesions by enhancing the level of inflammatory molecules such as COX-2, iNOS, and pro-inflammatory cytokines [20,39,40]. In addition, dermatitis inflammation induces ECM damage by inhibiting collagen biosynthesis and activating MMP activity, resulting in the suppression of skin regeneration in acne skin [41].

Hemp seed hexane extracts used in this study were obtained by hexane extraction to yield oil fractions. Hemp seed hexane extracts contained high level polyunsaturated fatty acids including linoleic acid, oleic acid, cis-11-eicosenoic acid, and palmitic acid, γ-linolenic acid, arachidic acid, palmitoleic acid and heneicosanoic acid (Data not shown) in this investigation. Linoleic acid has been known to be a significant constituent of the extracellular lipid matrix of stratum corneum [42]. Moreover, sebum of acne-prone skin was deficient in linoleic acid [43]. In addition, linoleic acid and γ-linolenic acid possess anti-inflammatory and anti-microbial activities, which inhibit inflammatory responses via the inactivation of NF-κB and AP-1 [41]. Therefore, major components HSHE, including linoleic acid and γ-linolenic acid, might be contributable for the improvement of acne vulgaris.

In this study, HSHE treatment suppressed the induction of inflammatory enzymes iNOS and COX-2 and their products NO and PGE_2_ caused by *P*. *acnes* infection. The secretion of inflammatory cytokines IL-1β and IL-8 was also reduced by HSHE. In addition, HSHE inhibited the phosphorylation of IKK, IκB, NF-κB, p38, JNK, and ERK, regulating NF-κB and MAPK signal pathways in *P*. *acnes*-induced HaCaT cells. HSHE could promote collagen synthesis *in vitro* and inhibit MMP-9 activity. These results suggest that HSHE might be beneficial in aiding the skin regeneration process on inflamed acne lesions.

PPARγ, which is highly abundant in adipose tissue, is known to be activated by IGF-1, linoleic acid, and cholesterol, leading to lipogenesis in sebaceous glands [44]. The expression of PPARγ is inhibited by AMPK, a major regulator of glucose and lipid metabolism [44,45]. When AMPK is phosphorylated, it reduces the expression of sterol regulatory factor binding protein-1 (SREBP-1), which is responsible for fatty acid synthesis through the mammalian target of rapamycin (mTOR), inhibiting the fatty acid synthase (FAS) and acetyl-CoA carboxylase (ACC). AKT pathway, a sub-cascade of insulin signaling, is implicated in adipocyte differentiation and FoxO1 regulation. AKT-mediated phosphorylation of FoxO1, resulting in its translocation from nucleus to cytoplasm, blocks the gene transcription involved in glucose production as well as in lipolysis [16,46]. In this investigation, HSHE reduced the expression of SREBP1 and FAS by activating IGF-1-induced AMPK in sebocytes. Phosphorylation of mTOR was also inhibited by HSHE. In addition, phosphorylation of FoxO1 by activated AKT was reduced.

**Fig 10:**
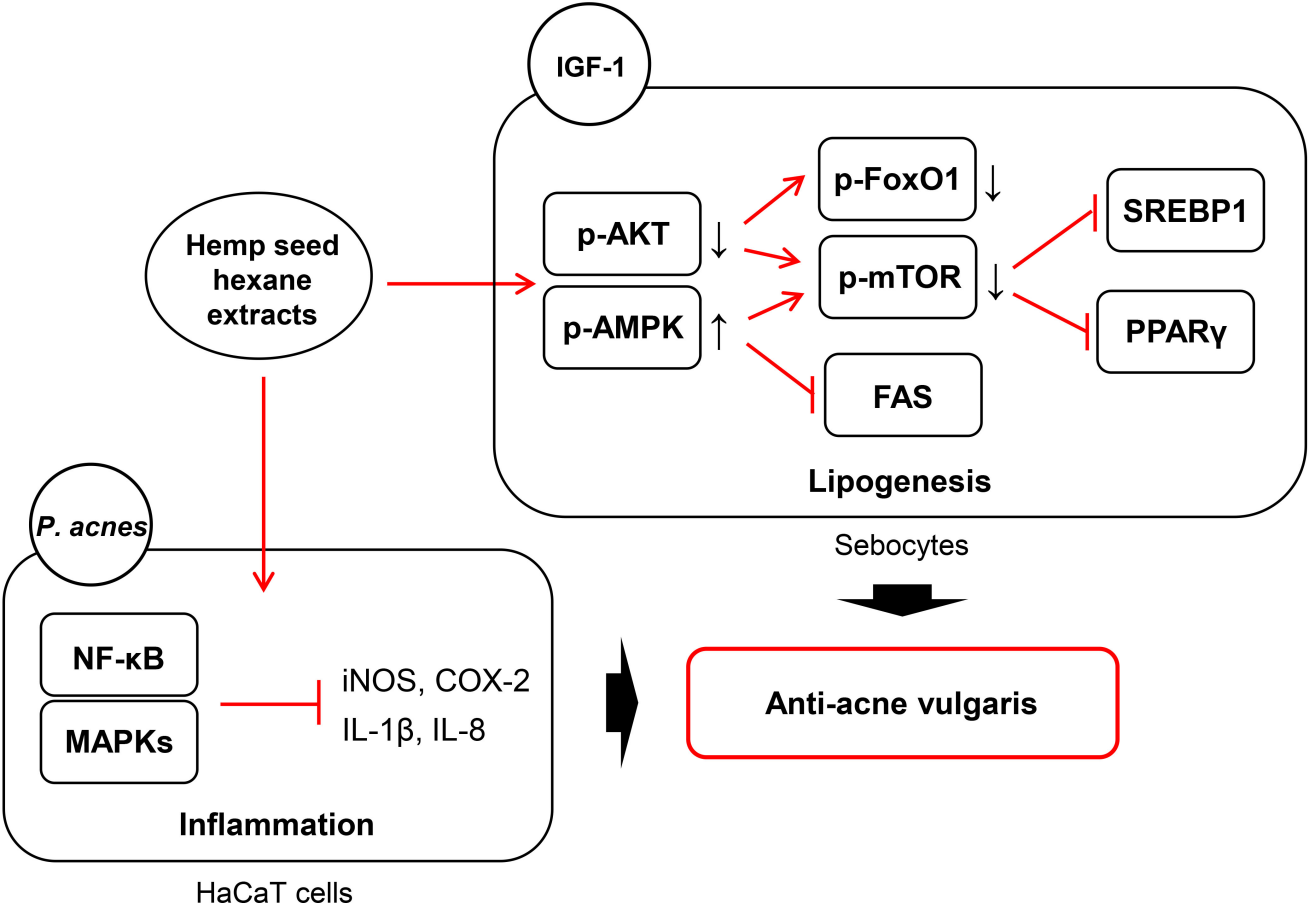
The underlying mechanism for anti-acne activity of hemp seed hexane extracts via anti-inflammation in *P*.*acnes*-stimulated HaCaT cells and anti-lipogenesis in sebocytes.

Our results demonstrate, for the first time, the underlying mechanism of anti-inflammation and anti-lipogenesis in sebocytes by HSHE and further unravel the mechanism of anti-acne vulgaris as depicted in Fig 10.

Clinical data showed that enhanced expression of COX-2 and PGE_2_ in acne skin was associated with increase in the secretion of pro-inflammatory cytokines. Moreover, sebocyte lipogenesis as well as inflammatory reactions could be modulated by PPAR signals, suggesting the interplay between lipo-inflammation and lipid signaling [47]. Thus, a strategy for blocking the inflammatory response as well as lipogenesis in sebocytes seems to be an ideal treatment for the improvement of acne lesions. Systemic treatment of acne patients with a 5-LOX inhibitor and PG inhibitor reduces the inflammatory acne lesions and the synthesis of sebum lipids[20,48]. Thus, HSHE seems to be the superior treatment for acne, overcoming the limitation of antibiotic resistance and retinoids, and the risk of side effects. In conclusion, this study confirmed the promising anti-acne activity of HSHE, evidenced by anti-*P*. *acnes*, anti-inflammation, anti-lipogenesis, and pro-collagen synthesis activities. Thus, HSHE could be utilized conventionally for the development of safe and effective anti-acne agents.
